# Thermal Mass Effect on the Solution Cooling Rate and on HIPped Astroloy Component Properties

**DOI:** 10.3390/ma15041434

**Published:** 2022-02-15

**Authors:** Unai Galech Napal, Miren Aristizabal Segarra, Borja Elguezabal Lazcano, Antonio Sivo, Iñigo Iturriza Zubillaga

**Affiliations:** 1 CEIT-Basque Research and Technology Alliance (BRTA), Manuel Lardizabal 15, 20018 Donostia-San Sebastián, Spain; maristizabal@ceit.es (M.A.S.); belguezabal@ceit.es (B.E.L.); iiturriza@ceit.es (I.I.Z.); 2 Tecnun, Manuel Lardizabal 13, Universidad de Navarra, 20018 Donostia-San Sebastián, Spain; 3 Department of Applied Science and Technology (DISAT), Politecnico di Torino, Corso Duca Degli Abruzzi, 24, 10129 Torino, Italy; antonio.sivo@polito.it

**Keywords:** Astroloy, HIP, Ni superalloys, metals and alloys, high-temperature alloys, thermal mass effect, cooling rate effect, gamma prime precipitation, gamma prime coalescence, heat treatments

## Abstract

Astroloy is a Ni-based superalloy with high-volume fraction of γ′, which gives high temperature properties but reduces its forgeability. Therefore, powder metallurgy manufacturing processes such as Near Net Shape HIPping are the most suitable manufacturing technology for Astroloy. However, NNSHIP has its own drawbacks, such as the formation of prior particle boundaries (PPBs), which usually tend to decrease material mechanical properties. The detrimental effect of PPBs can be reduced by optimizing the entire HIP processing route. Conventional HIP cycles have very low cooling rates, especially in big components from industry, and thus a series of post-heat treatments must be applied in order to achieve desirable microstructures and improve the mechanical properties. Standard heat treatments for Astroloy are long and tedious with several steps of solutioning, stabilization and precipitation. In this work, two main studies have been performed. First, the effect of the cooling rate after the solutioning treatment, which is driven by the materials’ thermal mass, on the Astroloy microstructure and mechanical properties was studied. Experimental analyses and simulation techniques have been used in the present work and it has been found that higher cooling rates after solutioning increase the density of tertiary γ′ precipitates by 85%, and their size decreases by 22%, which leads to an increase in hardness from 356 to 372 HB30. This hardness difference tends to reduce after subsequent standard heat treatment (HT) that homogenizes the microstructure. The second study shows the effect of different heat treatments on the microstructure and hardness of samples with two different thermal masses (can and cube). More than double the density of γ′ precipitates was found in small cubes in comparison with cans with a higher thermal mass. Therefore, the hardness in cubes is between 4 and 20 HB 30 higher than in large cans, depending on the applied HT.

## 1. Introduction

The continuous improvement in the performance of jet engines together with the need to reduce greenhouse gases emissions are associated with continuous research and development of the materials used in the aeronautical industry. In the past, steel alloys were used as the main component of engines, but nowadays other alloys such as nickel base superalloys have gained prominence. Nickel superalloys are widely used due to their magnificent properties, even at extreme temperatures, which are provided by the high-volume fraction of gamma prime (γ′; Ni_3_(Al, Ti)) precipitates that is usually above the 50% of the alloy [1,2,3,4,5,6,7,8,9,10,11]. These alloys usually include other elements such as Cr, Co, Mo and W in order to reinforce the matrix. However, the control of these alloying elements is crucial as they tend to form different carbides and strongly segregate in ingots, decreasing the materials’ machinability [5,8,9,12,13,14,15,16].

Astroloy is a well-known nickel-based superalloy that operates at temperatures up to 760 °C. Near Net Shape Hot Isostatic Pressing (NNSHIP) has been verified to be ideal for this nickel superalloy due to the avoidance of strong element segregation, and the decrease in the buy-to-fly ratio [17,18,19,20,21,22,23,24]. The NNSHIP of Ni superalloys has faced problems such as prior particle boundaries (PPBs), which are detrimental to the mechanical properties. The presence of PPBs is related to the concentration of carbon and oxygen in the original powder. Thus, the entire HIP value chain needs to be optimized in order to reduce their presence [17,21,22].

After HIP, heat treatments (HT) are applied in order to improve the material properties [22,25]. The first HT step is the solutioning, in which the precipitated γ′ is partially or totally dissolved in order to have the alloying elements available in the matrix for the formation of new precipitates, usually tertiary γ′ with smaller size. This is a key step since γ′ precipitates are coarse and irregular due to the slow cooling rates reached in industrial HIPping cycles [25,26,27,28,29]. During solutioning treatment, the component reaches a homogeneous temperature and a certain level of γ′ is dissolved based on the applied temperature. When the cooling process starts a thermal gradient is formed inside the specimen, producing different cooling rates and different γ′ precipitations, since the number of nucleation points is dependent on the cooling rate, which could affect the mechanical properties locally [10,25,30]. This thermal gradient is dependent on the component dimensions, being more pronounced as the size of the component increases. Higher cooling rates produce a smaller and more spherical γ′ due to the short time for coarsening. In this case, γ′ precipitation is driven by the nucleation more than by the coarsening, and this will provide a higher strengthening to the material at room temperature [10,25,30]. The cooling rates in different zones of large specimens can be estimated using numerical simulations.

After solutioning, more precipitation treatments are usually applied in order to promote the precipitation of more and finer γ′. These HTs involve a competition between the formation of new γ′ and the coarsening of the previous one by an Ostwald-ripening process, usually the tendency is to diminish the number of precipitates with the goal of reducing the interfacial energy between γ and γ′ [9,11,31,32,33].

**Figure 1:**
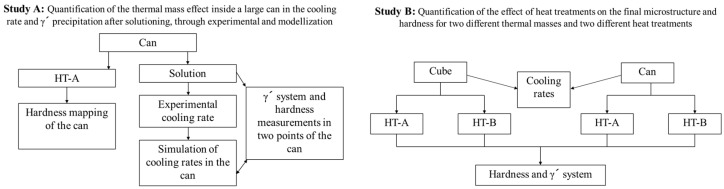
Graphical abstract of the steps performed in the paper.

In this work two studies are proposed: first of all, a quantification, through modelling and experimental characterization, of the effect of sample’s thermal mass, within a big can, in the cooling rate and γ′ precipitation after solutioning. These results have been discussed and related with the mechanical properties of the material. On the other hand, samples with two different dimensions (small cube and big can) have been HTed with two different HT and their microstructure have been analyzed and related with their mechanical properties. The following figure summarizes the work presented in this paper, see Figure 1.

This work presents a new approach for the quantification of the influence of cooling rate effect on Astroloy microstructure and properties considering the thermal gradient formed inside very large specimens, whereas other studies reported the cooling rate effect due to different environments.

## 2. Material and Methods

### 2.1. As-Received Powder

For the proposed study, two commercial gas-atomized Astroloy powders with a similar chemical composition were used. The chemical composition of the powders is reported in Table 1:

LECO CS-200 Series Carbon/Sulphur combustion analyzer (LECO Corporation, St Joseph, MI, USA) was used for carbon measurements, whereas a LECO TC-400 Series Oxygen/Nitrogen fusion analyzer (LECO Corporation, St Joseph, MI, USA) was used for oxygen measurements. ICP-OES (Inductively Coupled Plasma-Optical Emission Spectroscopy) (Agilent Technologies, Santa Clara, CA, USA) was used for the determination of non-interstitials elements with a Varian 725-ES.

**Figure 2:**
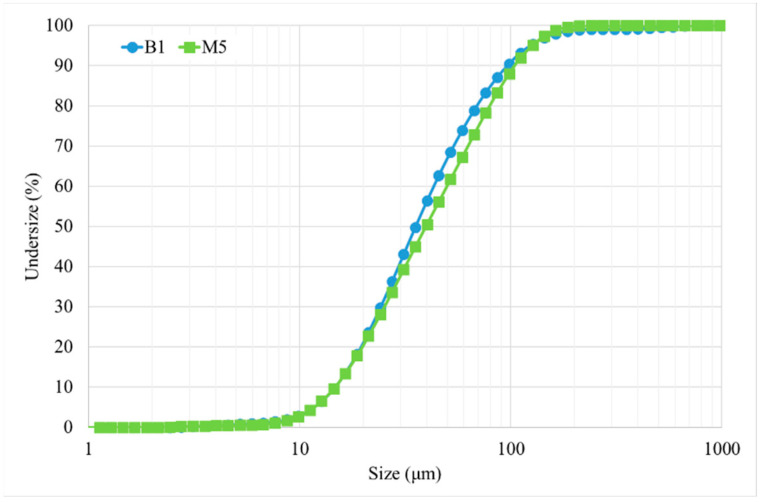
Particle size distribution of powders.

Both powders have similar particle size distribution between 10 and 150 μm, which was determined with a Mastersizer 3000 Malvern using the Hydro Module (see Figure 2).

### 2.2. HIP + HT and Samples Preparation

**In the first study (A),** two cans of 110 mm height × Ø 90 mm were filled with powder M5, degassed and HIPped at 1150 °C for 3 h at 103 MPa. The pressure and the temperature were increased and decreased at the same time, the heating rate of the process was 10 °C/min, whereas the cooling rate was 3 °C/min between 1150 °C and 400 °C. After HIP, one can was subjected to a unique HT, solution at 1115 °C/4 h and the other one was subjected to the standard HT, which is called HT-A. All the HTs followed a heating rate of 10 °C/min and were air cooled.

The HTs cycles were controlled with an external k type thermocouple linked to the external surface of the samples. However, the can which was HTed only with solution had two thermocouples in two locations (lateral and external upper) in order to assure a good control of samples temperature and the cooling rates, in order to have more reliable data for the modelling. The cycle was registered with an Agilent 34970A.

**Figure 3:**
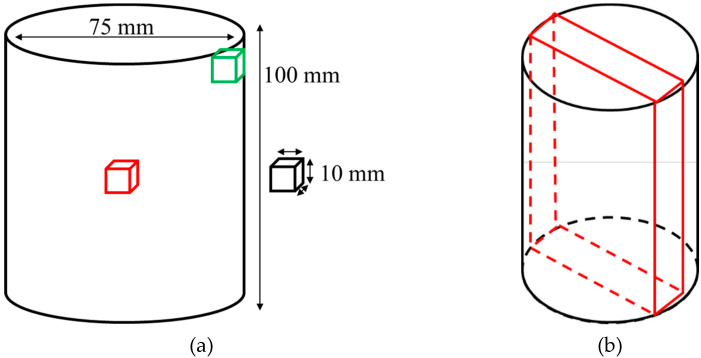
(**a**) Scheme of the can and cube dimensions, both after HIP, for study B. Additionally, the can microstructural analysis areas were pointed out, in red and green, for study A. (**b**) Extracted can slice for hardness mapping, study A.

On one side, a slice of the can with HT-A was extracted by EDM in order to have a big cross-section surface, where a hardness mapping study was carried out, see Figure 3b.

On the other side, a microstructural analysis and hardness measurements were performed on the inner and the external upper part of the can with only solution treatment, see Figure 3a. Samples for microstructural studies were cut and mounted in an acrylic conductive resin, grinded in 1200 grid papers and then polished with silica. Finally, samples were etched with kallings N°2 (5 g of CuCl_2_ + 100 mL HCl + 100 mL EtOH) reactant for 7 s.

**In the second study (B),** three cylindrical cans with the same dimensions were prepared with powder B1, following the same HIP cycle used in study A. Then, one can was cut into small cubes of 1 × 1 × 1 cm^3^ and two of them were taken for the study. From now on, these samples will be called “cubes”. The other two cans were maintained in their original state.

After HIPping, the first cube and first can were heat-treated with the standard procedure for Astroloy following the AMS 5852B, which has five steps, and from now on will be called HT-A. This HT involves solutioning at 1115 °C/4 h, stabilization 1 at 871 °C/8 h, stabilization 2 at 982 °C/4 h, precipitation 1 at 649 °C/24 h and precipitation 2 at 760 °C/8 h. On the other hand, the second cube and second can were subjected only to a solution and two precipitation steps, which will be known as HT-B treatment (see Table 2). In all cases, samples were always heated with a heating rate of 10 °C/min and air-cooled. In these HTs, a unique thermocouple was used, and it was linked to the external surface of the samples.

On one hand, mechanical and microstructural test specimens were extracted by EDM from the HIPped and HTed cans. On the other hand, cube samples were cut into two pieces, thus the middle of the sample could be analyzed for microstructural characterization. These samples followed the same preparation scheme as the previous ones for microstructural analysis.

### 2.3. Microstructural Assessment

The microstructural analyses of HIPped components were performed with a field emission gun-scanning electron microscopy (FEG-SEM) (ZEISS, Oberkochen, Germany) on a ZEISS SIGMA 500 equipment. In this work, backscattered images were taken due to the remarkable contrast between gamma prime (γ′) precipitates and matrix (γ). In particular, the images were taken mainly where grains showed square section of secondary γ′, in order to precisely calculate the particles dimensions. Furthermore, images have similar grey scale histograms in order to be as much precise as possible in the determination of γ′. Samples were mounted in a conductive acrylic resin and polished up to colloidal Silica, then they were etched with kallings N°2 reactant for 7 s.

Image processing was carried out via ImageJ software (National Institutes of Health, Bethesda, MD, USA). The program was used to enhance the grey-level difference between γ and γ′. Once both were perfectly distinguishable from each other, a black and white binary image was created, in which the black section represents γ′ precipitates. In all the cases, the same methodology was followed, using the same thresholding algorithm from the program trying to minimize the errors when the binary image is formed.

**Figure 4:**
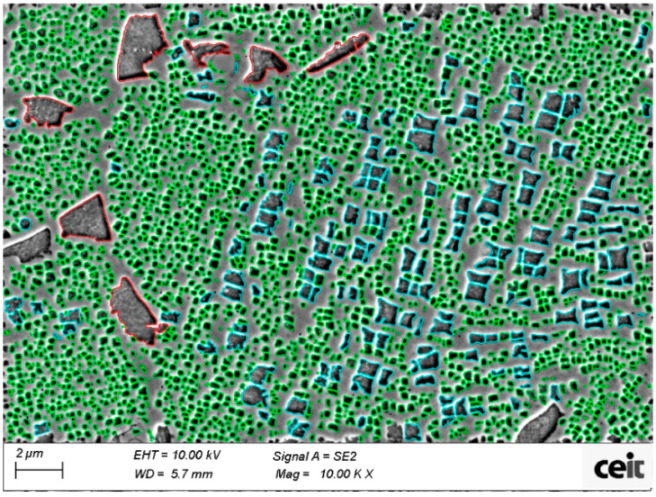
FEG-SEM image of a typical standard HTed Astroloy microstructure taken with the secondary electron detector. γ′ are coloured with red for primary γ′, blue for secondary γ′ and green for tertiary γ′.

The program evaluates the area of every γ′ precipitate and the total γ′ area; moreover, it is worth mentioning that this analysis was carried out on two-dimensional images, although reality is three dimensional, and it was assumed that results could be extrapolated from area fraction to volume fraction. In addition, γ′ precipitates can be classified into three types according to their size and shape: primary γ′, secondary γ′ and tertiary γ′, see Figure 4. Furthermore, γ′ precipitates that were in the images edges were removed from calculations in order to reduce possible source of uncertainty in the results of relative γ′ distribution and size calculations. However, there were included for the total γ′ quantity. Five images at 10 kX magnifications were taken in order to do a proper analysis (equivalent to 2579 μm^2^, with tens of thousands of precipitates involved).

**Figure 5:**
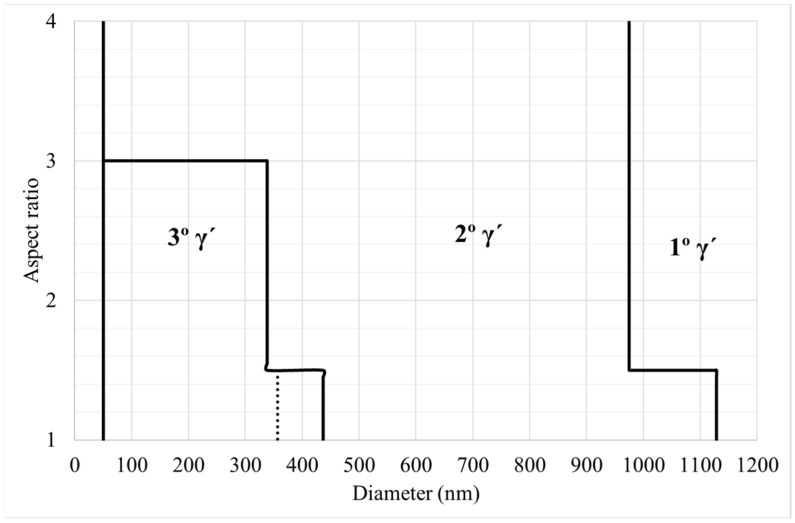
Segmentation parameters for γ′ precipitates in cans, the small change for cubes is reflected as dot points.

γ′ precipitates were classified as primary, secondary, and tertiary γ′, taking into account the size and aspect ratio of the precipitates. Figure 5 illustrates the parameters established for that classification in a graph. The effectiveness of classification has been checked by specific control images. In addition, particles with an area lower than 0.002 μm^2^ were classified as noise, in order to reduce possible uncertainty of the measurement. This value was found by an empirical approach. Cube samples have a smaller tertiary γ′, thus for them it was used a smaller tertiary γ′ size in order to improve the separation between tertiary and secondary γ′ (see Figure 5).

Size and area of the three γ′ types were measured using spherical approximation. This approach is good for the spherical tertiary and secondary γ′, but it is not the best approach for irregular shaped γ′ precipitates. However, the spherical approach was used in all the cases in order to have comparable results in size between the different types of γ′.

### 2.4. Hardness Evaluation

Brinell hardness measurements were carried out in a Universal Centaur Durometer (Metrol Centaur, Bilbao, Spain) with an applied force of 1.84 kN with a sphere diameter of 2.5 mm, Brinell HB 30. The hardness reported is the average of 10 indentations per sample. In contrast, for the hardness mapping a single measure was performed in every position, 80 measures were performed.

### 2.5. DSC Measurements

Differential scanning calorimetry (DSC) measurements were performed on a STA449 F3 JUPITER with a furnace of Rhodium capable of reaching 1600 °C on an alumina crucible. The DSC cycle has 3 steps. First, heating from 25 °C to 1450 °C. Then, a maintenance step of 15 min at 1450 °C. Finally, cooling from 1450 °C to 25 °C. The heating and cooling rates of the process were 10 °C/min. Every measurement was performed twice in order to obtain sharper peaks in the second cycle.

### 2.6. Simulations of Cooling Rates

The following heat balance equation has been used to model the cooling of the can after the heat treatment.

(1) $$\rho c_{p}\frac{\partial T}{\partial t}=\frac{1}{r}\frac{\partial }{\partial r}\left(k_{r}r\frac{\partial T}{\partial r}\right)+\frac{\partial }{\partial z}\left(k_{z}\frac{\partial T}{\partial z}\right)+\dot{Q}\;\;\;\;\;\;\mathrm{at}\;\Omega \;x\;t$$

where ρ, $c_{p}$ and $k_{r}$ are the material density, specific heat capacity and thermal conductivity, respectively. In addition, r and z represent the cylindrical coordinates, t is the time variable, T is the temperature variable and $\dot{Q}$ represents the heat source terms.

In the present case study, the parameters of density, specific heat capacity and conductivity in the desired temperature range are available for both the can material (mild steel) and the Astroloy.

The finite element technique has been used for the numerical solution of the above equation. However, the response of the finite element model is conditioned by the boundary conditions. In this case, they are defined by convection and radiation as shown in the following equations:

(2) $${\dot{q}}_{h}=-h\left(T-T_{r}\right)\;\;\;\;\;\;{\mathrm{at}\;\Omega }_{b}\;x\;t$$

(3) $${\dot{q}}_{r}=-\varepsilon \sigma \left(T^{4}-T_{r}{}^{4}\right)\;\;\;\;\;\;{\mathrm{at}\;\Omega }_{b}\;x\;t$$

where h represents the convection parameter, ε is the emissivity, σ and $T_{r}$ are the Stefan–Boltzmann constant and room temperature, respectively.

**Figure 6:**
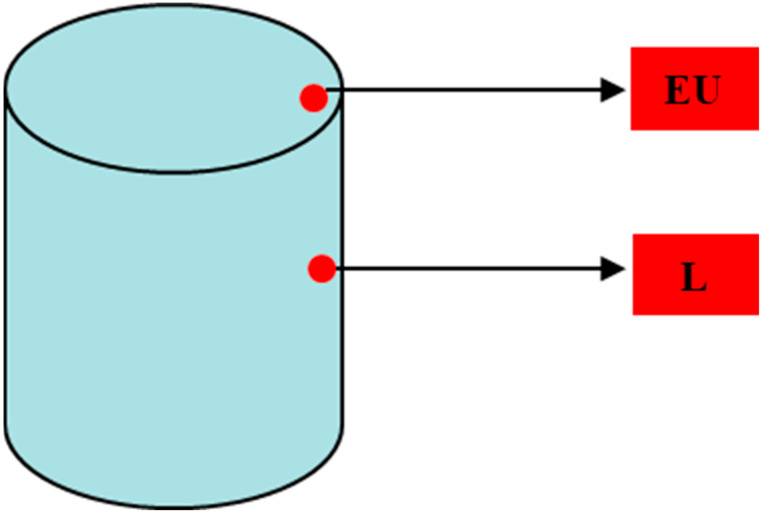
Fourth can thermocouple locations.

As the convection and emissivity parameters are not known, two thermocouples have been welded to the can, from which the temperature is measured every second during the cooling. The two thermocouples were fixed machining a small hole of a depth of 3 mm in both points. A schematic picture can be seen in Figure 6:

For the calibration of the numerical model (determination of the convection and emissivity parameters) the evolution of the temperature measured with the thermocouples has been used. For this purpose, a function (Equation (4)) has been defined, which measures the error between the evolution of the temperatures in the numerical model and those measured experimentally.

(4) $$f_{\mathrm{error}}=\overset{N}{\underset{i=1}{\sum }}{\left(T_{i}^{\exp}-T_{i}^{\mathrm{num}}\right)}^{2}$$

where *N* represents the number of measured points. For the minimization of this error function, an algorithm based on gradient descent has been used, which returns the convection and radiation parameters required to obtain the evolution of the temperatures measured in the thermocouples.

## 3. Results

The results have been divided in two studies. First, study A, where the can produced with powder M5 was analyzed in order to fill the model after solutioning, so that their microstructure and hardness could be related to the cooling rate. Additionally, the solutioning cooling rate influence on the final properties after a full standard HT (HT-A) has been assessed. Secondly, study B, where the effect of thermal mass on the microstructure and hardness of two samples of different dimensions with two different HTs were quantified; this was performed with powder B1. Although two different powders have been used, their properties are very similar, and they are not expected to behave differently.

### 3.1. Study A, Simulation Model

#### 3.1.1. DSC Analysis

**Figure 7:**
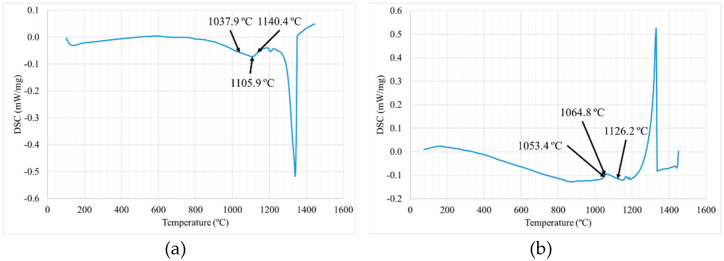
DSC of Astroloy B1 powder for (**a**) heating and (**b**) cooling.

DSC measurements were made on Astroloy powder B1 in order to obtain the ranges of temperatures of dissolution and precipitation of γ′ during the second cycle (see Figure 7).

During heating, material does not suffer any important microstructural change until γ′ dissolution between 1037.9–1140.4 °C (with the maximum peak at 1105.9 °C). In this range γ′ precipitates are dissolved, leaving more γ′ forming elements in the matrix for further precipitation during HTs. Other authors have also reported similar γ′ dissolution ranges in nickel-based alloys [10,25]. On the other hand, the precipitation range during cooling was determined to be between 1053.4 and 1126.2 °C, with the peak at 1064.8 °C. In this work, the solution was performed at 1115 °C, where γ′ that was present in the as-HIPped sample is mainly dissolved in this step. However, during the cool down from 1115 °C to 1053.4 °C, an important amount of γ′ can start to precipitate again.

#### 3.1.2. Solutioning Cooling Process for Simulation

**Figure 8:**
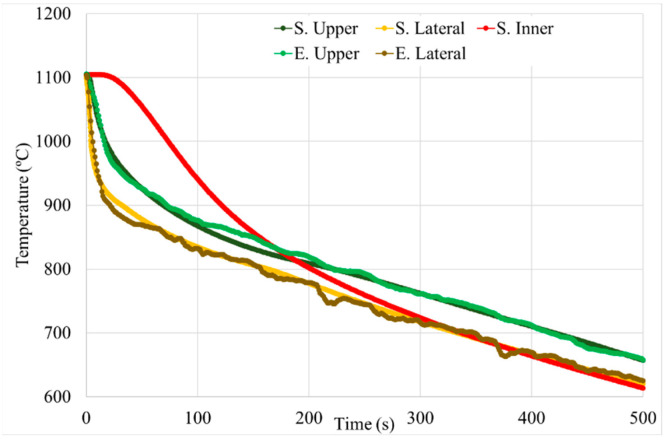
Solution cooling process of the powder M5 can, experimental (E) and simulated results (S).

A dedicated can was manufactured with powder M5 in order to collect data to fill a cooling rate model and validate the simulation. This can was subjected only to the solution step. Two thermocouples were connected to the can, as shown on Figure 6. The obtained data are shown in Figure 8. The cooling process was registered until 640 °C and the temperature was used in order to fill the model.

Simulation process curves fit well with the original ones (see Figure 8). The cooling rates of the three studied areas were obtained from these curves. The cooling rates were calculated from the initial temperature until 1053.4 °C for the external parts, although the cooling process of the inner part has a delay in time in comparison with the external parts. This is due to the cooling effect of the environment in the most external layers of the can. As a consequence, for the inner part, the derivative of the temperature with time was studied, thus the exact initial point was determined. (The results are gathered in Table 3.) For comparison, the fact that the γ′ precipitation range varies slightly with the cooling rate was omitted [30].

As it can be seen in Table 3, the experimental and simulated cooling rates are very similar. Therefore, the model was considered validated and the simulated cooling rates have been used in this work in order to have coherent results between the external cooling rates and the inner one. The inner part of the can has a different thermal profile compared with the outer, due to the different heat transport phenomena. The external part of the can is in contact with a cold fluid, the air, so the temperature difference is very high, and this increases the heat flux, but the inner part is in contact with more Astroloy, at high temperature, in all the directions, which produces lower temperature differences and a lower flux of heat.

#### 3.1.3. Hardness Measurements after Solutioning

**Figure 9:**
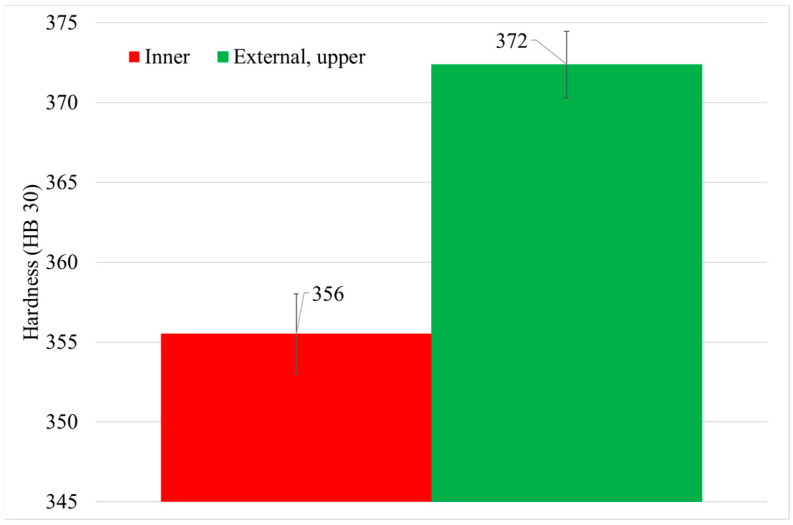
Hardness values in the inner and external part of the can.

Hardness measurements were carried out after solutioning, in the inner (middle) and external upper part of the can (see Figure 6). The results show a variation of 16 HB 30 between the inner and the external upper part of the can (see Figure 9). This difference is related to the cooling rates differences suffered during cooling of the solutioning stage.

#### 3.1.4. γ′. Quantification and Classification after Solutioning

**Figure 10:**
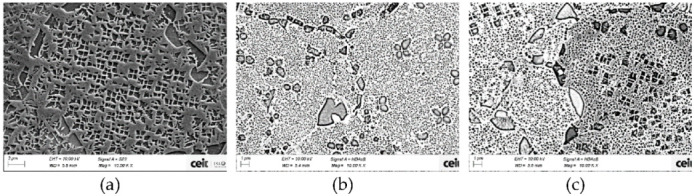
Microstructure of (**a**) as-HIP can (B1 powder) and solutioned can (M5 powder) in two areas (**b**) external upper part and (**c**) inner part.

Microstructures of inner and external areas were analyzed in order to understand the hardness variation found above. In this case, gamma prime analysis was focused only on tertiary γ′ since it is the main strengthening agent after solution treatment. Tertiary γ′ extensively changes from as-HIP state to solutioned state (Figure 10).

**Figure 11:**
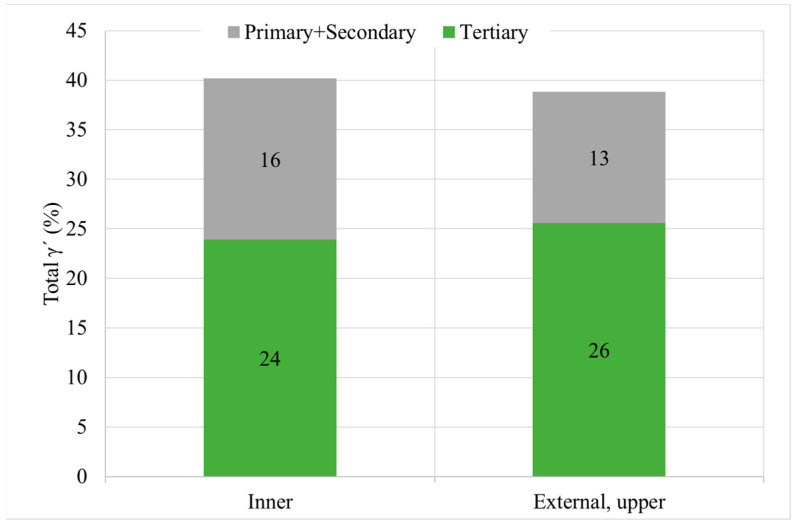
γ′ quantitative metallography after solution treatment of the samples including the general content and total area percentage for the inner and external upper part.

**Figure 12:**
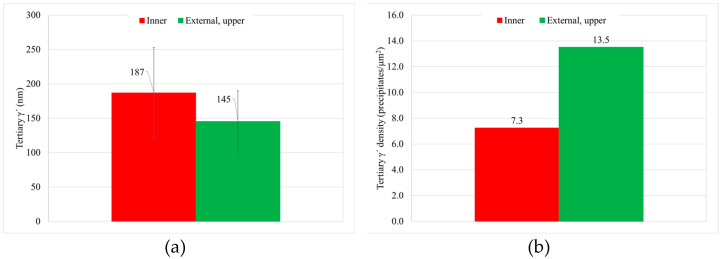
Tertiary γ′ (**a**) size and (**b**) density of precipitates after solution treatment in the inner and external upper part of the can.

From the quantitative metallographic analyses, it can be deducted that both areas have similar overall γ′ content and similar γ′ distribution (Figure 11). However, there is an important change in the tertiary γ′ size and density of precipitates. Tertiary γ′ size is coarser in the inner part than in the external part, whereas the number of precipitates is inversely proportional (see Figure 12). These microstructural differences are marked by the precipitation rate during the cooling process.

#### 3.1.5. Hardness Mapping after HT-A

**Figure 13:**
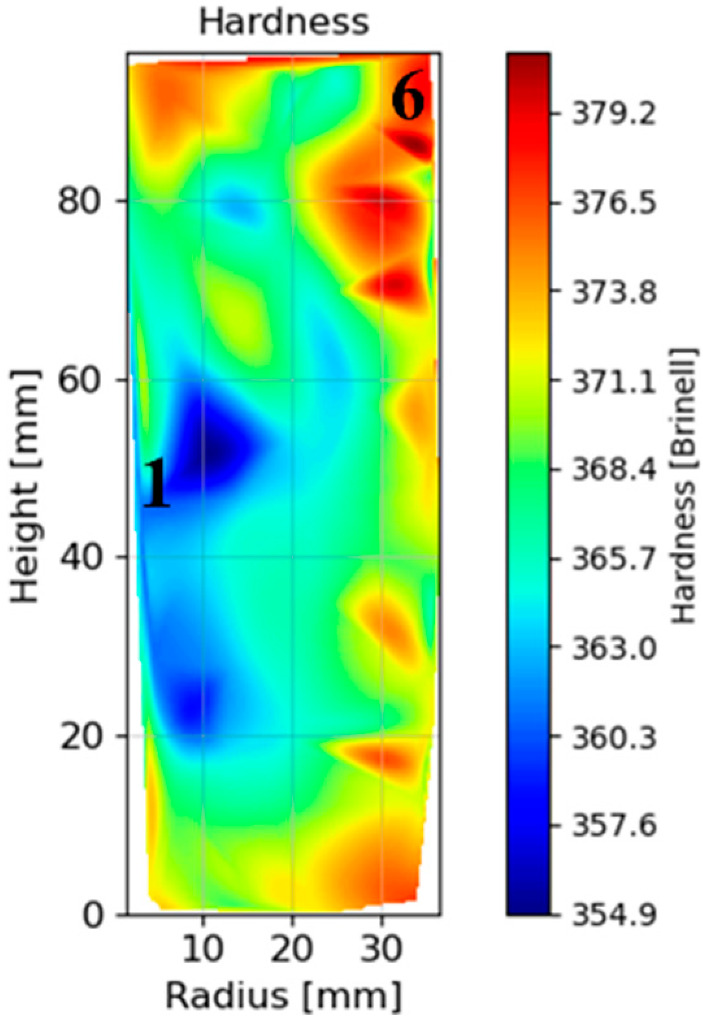
Hardness thermal mapping after HT-A, inner (1) and external upper (6) areas are marked.

After HT-A, hardness measurements were carried out in a central slide of the can. A hardness map of the whole surface was performed obtaining information from 80 points. The results show a variation in the hardness level between the inner and the external upper part of the can, areas 1 and 6, respectively (see Figure 13). In the mapping analysis, a unique indentation was performed in every point, the exact position of the indentation was measured in every case.

### 3.2. Study B, Can and Cube Samples

#### 3.2.1. Cooling Rate

**Figure 14:**
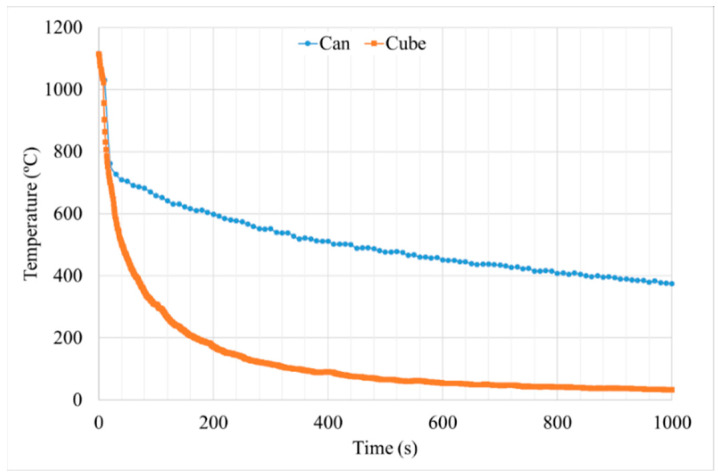
Temperature evolution during the solution cooling process of cube and can.

As it was mentioned above, temperature evolution in the HTs cycles was measured with an external thermocouple. The cooling rate in the surface of both samples is high at the beginning of the cooling step. Then, both samples continue the cooling process with a lower cooling rate, but the cube sample temperature decreases much faster than in the can. This tendency is repeated for all the steps, but it was included only the solution step of the HT-A sample as an example (see Figure 14). This is related to the sample’s thermal mass: as sample size increases, the mass that is needed to be cooled down increases, taking more time for the cooling process. As it was mentioned previously, the cube sample has a volume of 1 cm^3^, whereas the can volume has approximately 440 cm^3^ of Astroloy after the HIP process. Therefore, the can sample cools down much slower than the cube. The variation of the temperature with time for the can was recorded in its lateral area.

From HT-A samples the average cooling rate between 1115 °C and 400 °C were calculated in order to maintain the same temperatures range controlled, as in the HIPping cycle. The cube sample has a cooling rate of 11.2 °C/s, whereas the can has 0.8 °C/s, which indicates the huge difference between those samples in terms of cooling speed (see Table 4).

#### 3.2.2. Hardness Determination

**Figure 15:**
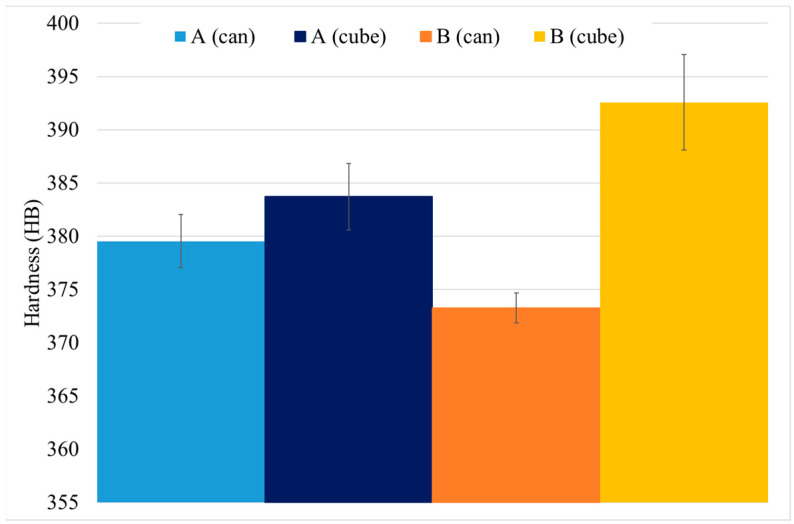
Hardness measurements of HT samples.

As expected, HTs increase the material hardness: 317 ± 8 HB 30 for as-HIP and above 370 HB 30 after HTs. However, this increase depends on the material’s size or mass and performed HTs. As it can be seen in Figure 15, there are small differences in hardness due to those effects, cube samples have higher hardness. Moreover, the difference in hardness for HT-B sample is higher than in HT-A.

#### 3.2.3. γ′. Determination

**Figure 16:**
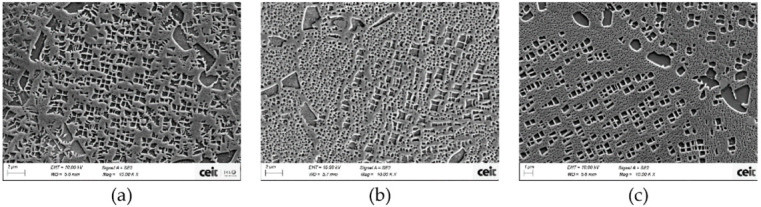
Astroloy microstructure revealed with kallings N°2 (**a**) after HIP and after HT-A for (**b**) can and (**c**) cube.

After HTs the microstructure has changed from a predominant microstructure of secondary γ′ precipitates in As-HIP sample to a majority of tertiary γ′. Moreover, can samples have a coarser secondary and tertiary γ′ compared with cube samples. In particulalr, tertiary γ′ have grown notoriously (see Figure 16). The same effect is also noticeable with HT-B samples.

**Figure 17:**
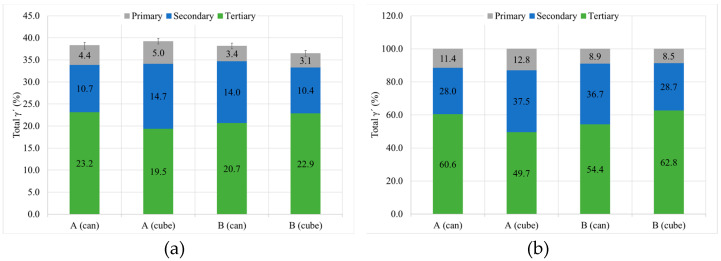
γ′ properties of the samples with HT-A and HT-B for the can and cubes, including (**a**) the general content and total area percentage and (**b**) relative distribution.

**Figure 18:**
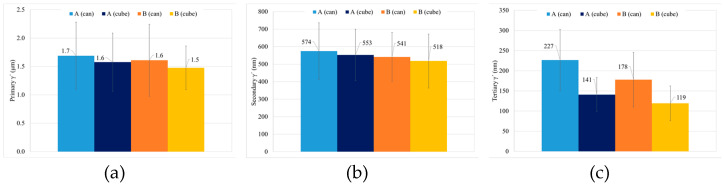
γ′ size by population of the samples with HT-A and HT-B for the can and cubes, (**a**) primary, (**b**) secondary and (**c**) tertiary.

**Figure 19:**
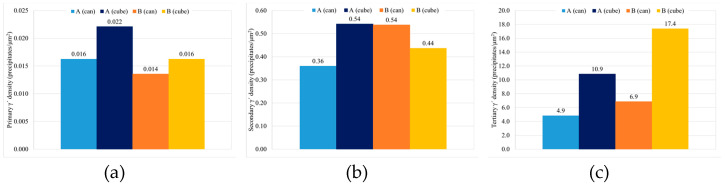
γ′ density of precipitates by population (**a**) primary, (**b**) secondary and (**c**) tertiary.

Total γ′ content of the four studied samples is similar, with a small decrease for samples with HT-B treatment, and tertiary γ′ is the more numerous strengthening precipitate in all the samples (Figure 17). In terms of γ′ precipitate size, can samples present larger precipitates than cubes. On the other hand, for samples with the same dimensions, material with HT-A treatment presents bigger precipitates than samples with HT-B treatment (Figure 18). Usually, when the precipitates are coarse their density is lower. This effect is very noticeable in the samples with similar overall γ′ content, where coarse tertiary γ′ precipitates show lower density than those which are thinner (see Figure 16b,c, Figure 18 and Figure 19).

## 4. Discussion

The discussion has been divided in the above mentioned two studies: Study A and Study B.

In study A, the relationship between the different solution cooling rate in several points of a large Astroloy component with the microstructure and hardness was analysed in as-solutioned material. In addition, hardness results of as-solutioned material were compared with the hardness of full heat-treated (HT-A) material.

In study B, two Astroloy samples with different dimensions, and thus different thermal mass, were studied in terms of microstructure and hardness. This study was carried out onto materials with two different heat treatment procedures, HT-A and HT-B.

### 4.1. Study A

#### 4.1.1. Relationship between Cooling Rate, Hardness and γ′ Microstructure after Solutioning

Solution cooling rate simulation fits very well with the experimental cooling rate data (Figure 8). Thus, measured hardness values, in specific points of the can, were related with simulated cooling rates (Table 5). The results showed that hardness increases from the inner part of the can to the external part (Figure 9). These differences in cooling rate provoke a clear change in the tertiary γ′ size and density due to the kinetics process that takes place during the cooling process (Table 5).

As the cooling rate increases, γ′ particle formation is slightly shifted to lower temperatures. If the cooling rate is too high, material could cool down below γ′ precipitation temperature, which could produce local alloying elements (Ni, Ti, Al) supersaturation due to a more restricted element diffusion. Lower precipitation temperatures produce a faster nucleation rate of γ′ due to the shorter time for precipitation, and the lower diffusion rates reduces the element movement in the matrix, which prevents the extreme coarsening of the particles. As a result, a very local precipitation of small γ′ particles in a huge amount would be formed. This local γ′, Ni_3_(Al, Ti), precipitation occurs at external areas where the cooling rate is extremely fast and γ′ precipitation is driven mainly by nucleation instead of by coarsening [11,30,34].

However, in the inner part, the process starts by forming an initial amount of γ′ precipitates and continues with a lower cooling rate than in the previous case. As a result, the alloying elements have a higher diffusion rate, and the matrix is less supersaturated since the particles have more time to precipitate. As a consequence, a competitive mechanism between the formation of new γ′ particles and the growth of those initial ones takes place. Considering that the precipitation time is longer due to the lower cooling rate, as shown in Table 3, the initial particles will tend to grow and even a small quantity of coalescence between particles could occur by an Ostwald-ripening mechanism in order to reduce the interfacial energy between γ and γ′. If this point is reached the coarser γ′ precipitates could lose their spherical shape [10,25,26,30,31,32,34,35].

Figure 10 shows the microstructure of the two areas with different cooling rate. The inner part, with a slower cooling rate, shows a 42 nm coarser tertiary γ′ in average size and a decrease in the density of precipitates of nearly the half (see Table 5). This behavior is consistent with the lower hardness, since bigger precipitates strengthen less the material [25,30,34].

The total amount of primary and secondary γ′, which is mostly related to the undissolved γ′ during solutioning, does not change significantly from the inner to the outer part. In the present work, the primary and secondary γ′ dissolution process is limited by the solution temperature of 1115 °C [10].

#### 4.1.2. Solutioning Cooling Rate Effect on the Final Hardness of a Full HTed Component

The effect of cooling rate, after solutioning, on the microstructure was confirmed and evaluated. In addition, it has been studied if this cooling rate effect on material properties remains after a full standard HT, HT-A.

For this reason, a hardness mapping on the whole surface of a central slice was performed, after HT-A. The hardness values obtained were represented against the cooling rate after solutioning. In this case, the cooling rate was obtained between the initial temperature and 1000 °C, in order to have the assurance of being out of the γ′ precipitation range after solutioning. Moreover, points 1 and 6 were marked, since both locations were discussed previously.

**Figure 20:**
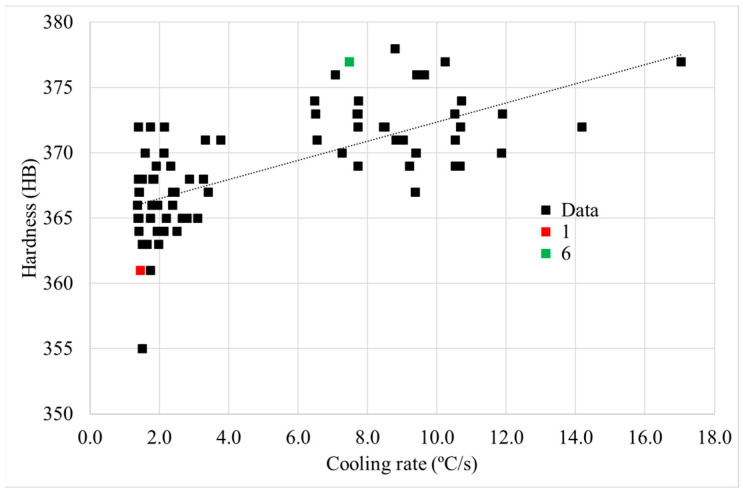
Relationship between solutioning cooling rate and hardness after HT-A.

Figure 20 represents the relationship between cooling rate after solutioning and hardness after a full HT. Despite the important scattering of the hardness mapping results, a slight relationship was found: as the solutioning cooling rate increases, the final hardness of the material increases. Thus, although this relationship was clearer in as-solutioned samples, the effect of solution cooling rate remains after a full HT process. After solution treatment, the γ′ size is much thinner at higher cooling rates due to the lower time for coarsening [30,36]. After performing the rest of the stabilization and precipitation HT, this γ′ tends to coarsen [9,33,35], and thus the hardness difference between the inner and outer part decreases, but still remains slightly different between the two parts of the material. The mean values of the two groups of points were obtained: 366 ± 3 HB 30 for the areas with lower cooling rate, and 372 ± 3 HB 30 for the faster cooling rates in the external area. Therefore, the hardness variation between inner and external areas has decreased from 16 HB 30, after solutioning (Figure 9) to 6 HB 30 after full heat-treated (HT-A) material.

### 4.2. Study B

Effect of Samples Dimensions and HTs in Final Microstructure and Mechanical Properties, Cube and Can Samples.

Cooling rate effect on microstructure was verified and evaluated after solutioning. Additionally, it was noted that these differences tend to reduce with the following HTs but are still present.

In this study, two samples of very different dimensions were studied, thus the cooling rate effect on microstructure could be more extremely seen. Moreover, the effect of following HTs on those samples was studied. Therefore, the evolution of the γ′ system could be followed on two different thermal masses.

**Figure 21:**
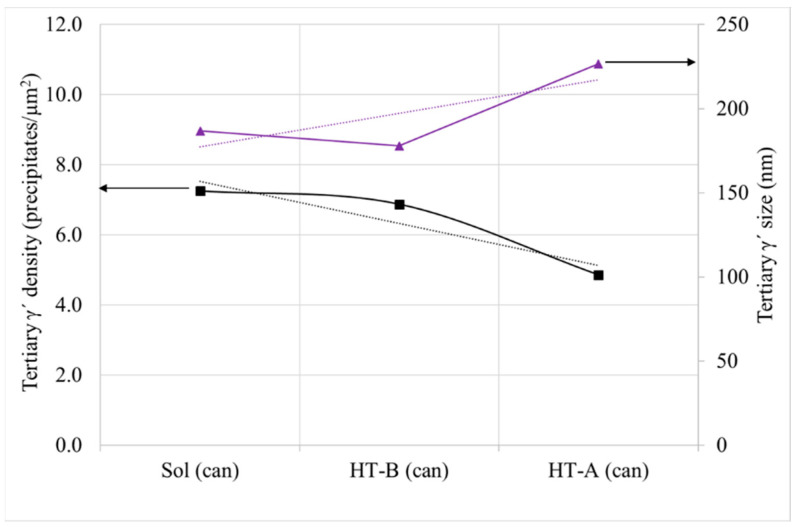
Relationship between tertiary γ′ density, size and following HTs (Sol: Solution, HT-B: Sol + P2 and HT-A: Sol + S1 + S2 + P1 + P2).

In the previous study, the effect of cooling rate on the microstructure was checked, but the cooling rate between those two areas was not extremely different. In this case, the cube sample has a volume of 1 cm^3^ and can 450 cm^3^, thus the cooling rates differences are even higher (see Table 4). Furthermore, it has to be considered that during HTs, particles are going to coarsen and the density of them will be reduced, thus the interfacial energy between γ and γ′ could be reduced (see the example of the cans (Figure 21)). This effect is more extreme for tertiary γ′ [9,15,29,33,35]. The inner part of the can was taken for comparison, since the majority of the can follows a slower cooling rate than the external part.

**Figure 22:**
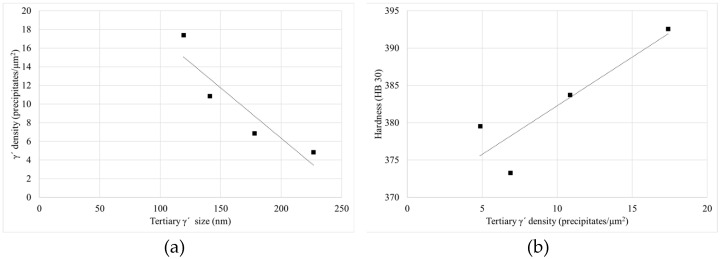
Relationship between (**a**) tertiary γ′ density—size and (**b**) tertiary γ′ density—hardness.

As it can be seen for the two studied dimensions and both HTs there is a quite clear relationship between the density of tertiary γ′ precipitates and their size. As long as the size increases the density of precipitates decreases, for a similar overall γ′ content of 38%. This happened also inside the can, as it was explained previously. This is due to the coarsening mechanism that takes place during the HTs, as long as the HTs steps or duration increase the coarsening of particles is more extreme, so the interfacial energy between γ′ and γ′ can be reduced (see Figure 22a) [9,10,15,28,33].

The γ′ system is mainly responsible for the increase in the hardness of the alloy, which increases after HTs. This is due to the precipitation of tertiary γ′, which provides higher strength to the material. Furthermore, the samples with the smallest precipitates, and the highest density of them, give the higher level of hardness (see Figure 18 and Figure 22b) [25,30,34]. Cube samples have a higher amount of precipitates due to the extreme cooling rates (see Figure 14 and Figure 19c).

## 5. Conclusions

In this work, the cooling rate was studied through the effect of thermal mass gradients inside massive components. Other authors also studied this effect by changing the cooling environment after solutioning (oil, water and so on). However, in this study the atmosphere was maintained and the cooling rate differences are only due to thermal mass. Furthermore, the effect of HTs on the final sample microstructure and hardness was assessed, also after a full HT-A. As a result, the effect of thermal mass gradients within large components (such as engine casings) can be quantified and evaluated if it compromises final component behavior. Moreover, the effect of sample dimensions and HTs on γ′ was studied through two samples of very different dimensions, cubes and cans. The main conclusions are summarized:

1. First, after solutioning, a correlation between cooling rate and hardness inside big components has been confirmed. Moreover, this has been proved to be related to the variation in γ′ system. Higher cooling rate leads to a higher density of tertiary γ′ precipitates with a lower size. The external area of the can has an 85% more of tertiary γ′ precipitates with a 22% lower size than the inner part, which leads to an increase of 16 HB 30. However, the external part of the component that suffers this high cooling rate is small.
2. After a full HT, the hardness differences in the component tend to reduce, but a small difference is maintained. The hardness differences between the external and the inner part of the can are reduced from 16 to 6 HB 30. Therefore, it would be recommendable to have a track of the place that samples are extracted inside big components. This effect could be much higher in gigantic parts, and could be the subject of a future work.
3. HTs after solutioning provokes the increase in the volume fraction of γ′, mainly through coarsening and coalescence of the previous existent precipitates, as can be seen in the decreasing population density of tertiary γ′. In the case of cans, from solutioning to a full standard HT (HT-A), the density of tertiary γ′ precipitates decreases by 33%, whereas their size increases by 21%. These means of precipitation are related to the obtention of the lower energy state in the material, reducing the interface energy between γ and γ′.
4. Cube samples have much higher cooling rates than cans due to their much lower thermal mass, which produces a much finer microstructure. As a result, cube samples have double the number tertiary γ′ precipitates with a reduction in size of at least 33%, which increases cube sample hardness by a minimum of 4 HB 30.

## Figures and Tables

**Table 1:** Chemical composition of Astroloy powder (wt.%).

Powders	Ni	Co	Cr	Mo	Al	Ti	Fe	B	C	O
B1	55.3	17.0	15.3	4.8	3.9	3.6	0.05	0.026	0.015	0.015
M5	55.3	16.8	15.1	5.0	3.9	3.8	0.06	0.026	0.020	0.009

**Table 2:** HTs steps per type of HT.

HT	Solution	S1	S2	P1	P2
A	X	X	X	X	X
B	X				X

**Table 3:** Experimental vs. simulated cooling rates for the can from 1115 °C to 1053.4 °C.

Area	Data	Cooling Rate (°C/s)
Upper	Experimental	5.3
	Simulation	6.4
Lateral	Experimental	16.6
	Simulation	19.0
Inner	Simulation	1.3

**Table 4:** Average cooling rate between 1115 °C and 400 °C after solutioning for cube and can HT-A samples.

Sample	Cooling Rate (°C/s)
Can	0.8
Cube	11.2

**Table 5:** Inner and external upper areas information, after solutioning.

Location	Cooling Rate (°C/s)	Hardness (HB 30)	Tertiary γ′ Density (Precipitates/μm^2^)	Tertiary γ′ Size (nm)
Inner (1)	1.3	356 ± 3	7.3	187 ± 66
External upper (6)	6.4	372 ± 2	13.5	145 ± 44

## Data Availability

All the data and results supporting this research paper are already presented within this publication.
